# A preliminary qualitative exploration of perceived barriers and facilitators prior to implementation of a pressure injury prevention program in home care: perspectives of care managers in Japan

**DOI:** 10.20407/fmj.2026-001

**Published:** 2026-09-25

**Authors:** Keisuke Iwase, Hironori Tsuzuki, Masushi Kohta, Koji Harima, Junko Sugama

**Affiliations:** 1 Faculty of Nursing, School of Health Sciences, Fujita Health University, Toyoake, Aichi, Japan; 2 School of Health Science, Department of Nursing, Toyohashi Sozo University, Toyohashi, Aichi, Japan; 3 Research Center for Implementation Nursing Science Initiative, Fujita Health University, Toyoake, Aichi, Japan; 4 Sapporo Keijinkai Rehabilitation Hospital, Sapporo, Hokkaido, Japan

**Keywords:** Care manager, Implementation research, Long-term care, Pressure injury, Risk assessment

## Abstract

**Objectives:**

This study aimed to explore care managers’ (CMs) perceptions of potential barriers and facilitators to implementing a pressure injury (PI) prevention program within Japan’s home care system.

**Methods:**

A pilot qualitative study was conducted using the Consolidated Framework for Implementation Research (CFIR) to guide data collection and analysis. Semi-structured interviews were conducted with five CMs employed at a home-based long-term care support facility (October 2022–March 2023). Two researchers independently coded the transcripts, reached consensus through discussion, and conducted member checking to enhance rigor.

**Results:**

A total of 32 facilitators and 32 barriers were identified across five CFIR domains. Key facilitators included the program’s perceived ease of use and its potential to enhance multidisciplinary communication. Frequently perceived barriers included uncertainty regarding the program’s effectiveness, insufficient knowledge and low perceived urgency regarding PI prevention among CMs and chief CMs, excessive workload, and limited resources. As participants had no prior experience implementing the program, these findings reflect anticipated perceptions rather than actual implementation experiences.

**Conclusions:**

This study provides preliminary observations regarding potential facilitators and barriers to implementing a PI prevention program in home care settings. Future research is required to further examine these perceptions and explore whether similar barriers and facilitators are observed in other settings and populations.

## Introduction

Rapid population aging is progressing globally as a result of declining fertility rates and increasing life expectancy. Japan has experienced a continuous rise in the aging rate.^1^ To reduce the demand for hospital beds, the Ministry of Health, Labour and Welfare has promoted a shift from a hospital-centered to a home-care-centered healthcare system.^2^ Pressure injuries (PIs) reduce the quality of life (QOL) of home care users and their families, and the risk of PI development may be heightened in home care settings where resources are limited compared with hospitals.^3–7^

Risk assessment scales are recommended as the first step in PI prevention.^8,9^ The Pressure Injury Primary Risk Assessment Scale for Home Care (PPRA-Home) was recently developed as a PI risk assessment tool designed for home care settings.^10^ In addition to serving as a risk assessment scale, the PPRA-Home is a core component of a multidisciplinary PI prevention program. The primary users of the PPRA-Home are care managers (CMs), who play a key role in promoting PI prevention in Japan’s home care system.

In Japan, CMs are a unique professional group established under the long-term care insurance system. They typically work at In-Home Care Support facilities, where they develop and evaluate care plans, coordinate services, and adjust care based on the physical and psychological conditions and preferences of their clients.^11,12^ To be certified as a CM, individuals must have at least 5 years of clinical experience in medical or social welfare professions and must pass a national examination; therefore, CMs have diverse professional backgrounds.^13^ The knowledge and attitudes of CMs toward PI prevention often differ by their backgrounds; those with social welfare training may struggle with assessment and problem analysis^14^ and may have less experience implementing PI prevention interventions compared with medically trained CMs.^15^

Implementation research focuses on how evidence-based interventions can be introduced and sustained in real-world settings.^16–18^ The widely used Consolidated Framework for Implementation Research (CFIR) highlights that implementation is influenced by factors at individual, organizational, cultural, and societal levels.^19,20^ Barriers to implementation include limited staff knowledge, heavy workloads, insufficient resources, and inadequate training, whereas clear institutional structures, improved organizational culture, better staff communication, and positive attitudes serve as facilitators.^21–23^ However, previous studies have primarily targeted nurses working in hospitals or long-term care facilities. To our knowledge, few studies have examined the perspectives of CMs involved in PI prevention in home care settings in Japan. As CMs act as central coordinators that integrate preventive care into individualized care plans, understanding their perspectives is essential to successful implementation. Given the limited existing evidence in this area and the lack of implementation experience among CMs, an exploratory approach was considered appropriate for this study. Therefore, this study aimed to explore CMs’ perceptions of the barriers and facilitators of implementing a PI prevention program in home care settings in Japan.

## Methods

### Study design

A pilot qualitative exploratory study was conducted from October 2022 to March 2023 at a single home care support center in Japan. An exploratory qualitative approach was considered appropriate to explore participants’ perceptions of implementation-related factors prior to program implementation. Ethical approval was obtained from the Institutional Review Board of Fujita Health University (Approval No. HM22-358).

### Reporting guideline

The reporting of the qualitative component followed the Standards for Reporting Qualitative Research (SRQR) checklist (Supplementary Material S1), developed by the Enhancing the QUAlity and Transparency Of health Research (EQUATOR) Network.

### Study population

The participants were CMs employed at a single facility. After obtaining approval from the head of the facility, the study purpose and procedures were explained to each CM, and written and verbal informed consent was obtained from all participants. Given the exploratory nature of this study, a purposive sample of five CMs was recruited to obtain detailed perspectives regarding potential PI prevention implementation barriers and facilitators. Participants were selected based on their availability and willingness to participate; therefore, the potential for sampling bias cannot be excluded. No additional inclusion or exclusion criteria were applied to the study.

### Description of the PI prevention program

The PI prevention program used in this study has been described previously.^24^ Because none of the participants had prior experience using the program, they received a 1-hour online orientation conducted by the researchers before data collection. The program consisted of two components:

#### 1) PPRA-Home risk assessment tool

The PPRA-Home includes eight binary (yes/no) items. A total score ranging from 0 to 8 is calculated by summing the scores of each item. Higher scores indicate a greater risk of developing PIs. The reliability and validity of the PPRA-Home have been confirmed in previous studies.^25,26^

#### 2) Checklist for multidisciplinary collaboration

This checklist is designed to assist CMs in gathering information on PI risk factors, identifying strategies to reduce these risks, and facilitating collaboration with other professionals.

### Data collection

An interview guide was developed based on the five CFIR domains^27^ (Supplementary Material S2). Semi-structured interviews were conducted using this guide, and additional probing questions were asked when necessary. As participants had no prior experience with the program, the interview questions focused on anticipated barriers and facilitators to implementation. Interviews were conducted by trained researchers with experience in qualitative research. All interviews were conducted in Japanese, audio recorded with participants’ consent, professionally transcribed, and checked for accuracy by the researchers. Each interview lasted approximately 30–60 minutes.

### Data analysis and coding

Data analysis and coding were conducted following the qualitative analysis procedures described by Saito et al.^28^ The procedure is briefly summarized here: 1) two researchers independently coded the data from interview transcripts according to the CFIR components; 2) the two researchers then compared the coded content and a consensus was reached; 3) a summary memo was prepared for each participant from the coded content; 4) to confirm the validity of the consensus, each summary memo was discussed and confirmed with a third researcher who was not involved in the coding process; 5) to improve the reliability of the results, the summary memo was provided to the participants, and their comments were obtained; and 6) the barriers and facilitators were explored and categorized in line with the CFIR constructs, after which a schematic was prepared to illustrate the relationships among the constructs.

The researchers had backgrounds in nursing and community health, which may have influenced data interpretation; reflexive discussions were conducted throughout the analysis to minimize potential bias. To enhance analytic transparency, data coding was systematically organized using spreadsheets, and the integration of codes was achieved through iterative consensus discussions among the researchers.

## Results

### Participant characteristics

Five CMs (one male and four female) participated in this study (Table 1). A total of 32 facilitators and 32 barriers to PI prevention program implementation were identified (Table 2).

### Overview of key themes

Among the identified factors, four recurring themes were identified across participants’ responses and multiple CFIR domains:

1) uncertainty regarding the program’s effectiveness,
2) heavy workloads and staff shortages,
3) limited knowledge and low perceived urgency regarding PI prevention, and
4) challenges in multidisciplinary communication.

These themes were derived through iterative coding, consensus discussions among researchers, and categorization of barriers and facilitators according to the CFIR framework. They represent recurring concerns expressed by participants regarding anticipated implementation challenges. A detailed mapping of facilitators and barriers across the CFIR domains is presented in Table 2.

### Intervention characteristics domain

Uncertainty regarding the program’s effectiveness emerged as a prominent barrier in the Intervention Characteristics domain. Participants expressed concerns about the lack of clear evidence and practical guidance for implementation (Table 2). By contrast, the program’s simplicity and its adaptability to existing PI risk factors were identified as key facilitators. However, participants also reported difficulties in sharing assessment results with other professionals and family members.

A representative comment included:

*“I don’t know how to share the PI assessment results with other professionals and the patient’s family members.”*

### Outer setting domain

In the Outer Setting domain, challenges in multidisciplinary communication and a lack of cooperation from patients and their families were identified as prominent barriers (Table 2). Alternatively, participants perceived that clearer evidence of the clinical effectiveness of PI prevention, along with policy improvements and expanded financial support, could serve as potential facilitators for the future implementation of the program. However, difficulties in communicating between CMs with different professional backgrounds, particularly those with social welfare training, were also highlighted.

Specifically, participants stated:

*“Some patients may be reluctant to implement the PI prevention program.”*

*“CMs with social/welfare backgrounds find it difficult to collaborate and communicate with medical professionals, and they often hesitate to contact them when any care or assistance is needed.”*

### Inner setting domain

Heavy workloads and staff shortages emerged as particularly prominent barriers in the Inner Setting domain (Table 2). Key facilitators included smooth workplace communication, consultation opportunities fostered by positive interpersonal relationships, and regular training. Participants also perceived that clear directives and leadership from supervisors, along with organizational incentives, may support program implementation. Conversely, several factors were described as significant barriers: chronic staff shortages, heavy workloads that hinder collaboration, and a lack of shared understanding regarding the priority of PI prevention. Additionally, the perceived low necessity of the program in facilities with few high-risk patients and incompatibility with existing assessment tools were also highlighted.

Specifically, participants stated:

*“A large number of individuals at our facility are at high risk of developing PIs. However, facilities with smaller numbers of such individuals are less likely to appreciate the need for the program.”*

*“Our facility has a specific method to assess PI risk. Incorporating PPRA-Home into the method can facilitate implementation of the program.”*

### Characteristics of individuals domain

In the Characteristics of Individuals domain, limited knowledge and low awareness of PI prevention emerged as prominent barriers (Table 2). By contrast, prior successful experiences with multidisciplinary collaboration, shared perceptions of PI prevention within the facility, and active communication among CMs were associated with enhanced self-efficacy and were perceived to support program implementation. Participants also reported additional barriers, including a lack of experience in consulting other professionals and heavy individual-level workloads.

Specifically, participants stated:

*“I think I have poor knowledge about PIs because my background is that of a social worker and not a medical professional.”*

*“Because CMs typically make solo visits to the homes of individuals requiring care, without accompanying medical professionals, it takes time and effort to share information with other professionals.”*

*“I believe that my successful experiences of PI prevention led to the successful implementation of the program.”*

### Process domain

In the Process domain, advanced planning and integration into routine practice were perceived as key facilitators for future implementation (Table 2). Participants suggested that planning and advance preparation may facilitate the implementation of the program. They also felt that conducting a pilot study before full-scale implementation and incorporating the program into daily work routines may support the ongoing practice of PI risk assessment.

Representative comments included:

*“It would be possible to conduct the program if the necessary preparations for its implementation are completed prior to home visits.”*

*“I believe that incorporating the program into my current work and using it on a regular basis will make it a habit.”*

## Discussion

This study provides preliminary observations regarding participating CMs’ perceptions of the barriers and facilitators of implementing a PI prevention program in home care settings. It should be interpreted as a preliminary qualitative exploration based on a small sample from a single facility rather than a comprehensive representation of CMs in Japan. Future studies may examine whether similar perceptions are observed in other settings and populations.

Through qualitative analysis of the perceptions of CMs, several themes regarding the implementation of a PI prevention program were identified. These included uncertainty regarding the program’s effectiveness, heavy workloads and staff shortages, limited knowledge and low perceived urgency regarding PI prevention, and challenges in multidisciplinary communication. These themes represent the perspectives expressed by participating CMs and should be interpreted within the context of this exploratory study. Some of the themes identified, including limited knowledge regarding PI prevention, workload-related constraints, and challenges in multidisciplinary communication, are similar to barriers previously reported among hospital clinicians and nursing home staff involved in PI prevention and management. Previous studies have identified that knowledge deficits, resource constraints, understaffing, and communication-related challenges are important barriers to PI prevention and guideline implementation.^29,30^ However, CMs in the present study also described issues that may be related to their specific role in coordinating care across multiple providers and settings.

Based on the themes identified in this exploratory study, we outline five considerations for future implementation. These considerations should be viewed as exploratory suggestions derived from participants’ perspectives rather than evidence-based recommendations. They may be useful for informing future research and implementation efforts in home care settings.

### Case selection

Case selection considerations were informed by heavy workloads and staff shortages identified in the Inner Setting domain, where participants described limited time and competing demands as barriers to the implementation of the program. To address these barriers, one potential consideration is to prioritize or limit the number of cases covered by the program. Japanese CMs manage many individuals who require care and support, and heavy caseloads per CM produce significant time constraints. While performing PI risk assessments for all individuals receiving home care is ideal, our findings suggest that an excessive workload is a substantial barrier to implementation. Further research may be needed to explore the appropriate criteria for case selection based on CMs’ perspectives, and observational approaches may be useful in assessing their efficacy. Despite the advantages of reducing CM workloads, this consideration inevitably limits the opportunities for risk assessment among at-risk individuals. Therefore, in addition to case selection, considerations to enhance CMs’ knowledge and awareness of PI prevention may also be important. These efforts may help ensure that high-risk cases are not overlooked.

### CM training

Considerations about CM training were informed by limited knowledge and low perceived urgency regarding PI prevention in the Characteristics of Individuals domain. In this domain, participants described how variations in CMs’ professional backgrounds led to differing perceptions of PI prevention, which may be barriers to program implementation. These findings may suggest that improving CMs’ knowledge and awareness regarding PI prevention may contribute to supporting program implementation.

CMs with social or welfare backgrounds may experience anxiety when managing cases as a result of their limited knowledge of medical conditions and procedures.^31^ A study reported that CMs’ knowledge of PI prevention was moderate but inadequate.^15^ The findings of this study are consistent with previous research, suggesting that the current knowledge and awareness of CMs regarding PIs may be limited. Additionally, heavy workloads may be associated with a lack of tension for change regarding PI prevention, highlighting the potential importance of educational interventions targeting CMs to enhance their knowledge and awareness. Educational programs for care providers with limited medical training may reduce the incidence of PI.^32^ Furthermore, e-learning and digital training programs improve knowledge, attitudes, and confidence in PI care among nurses.^33,34^ Collectively, these findings suggest that providing structured training on PI prevention at the time of certification or renewal may be beneficial. Tailoring training programs according to CMs’ professional backgrounds may also be useful.

### Leadership engagement

Leadership engagement considerations were informed by heavy workloads and staff shortages identified in the Inner Setting domain. In this domain, participants highlighted that chief CMs may hesitate to introduce new programs because of concerns about increasing the already substantial workloads of their staff. Such managerial concerns, combined with chief CMs’ administrative responsibilities, may act as barriers to organizational implementation.

These findings suggest that engagement of chief CMs may be an important factor in supporting implementation. While CMs are responsible for comprehensive care management for individual clients, chief CMs oversee daily operations as well as managing their own caseloads, indicating that their understanding and support may contribute to implementation at an organizational level. Previous studies have reported that clients managed by chief CMs may achieve better outcomes compared with those managed solely by CMs.^35,36^

Chief CMs often concurrently serve as administrators of home-based long-term care support facilities, which may make them particularly attentive to balancing workload and perceived benefits. Therefore, it may be useful to consider approaches that enhance their understanding of the potential value of the program and its integration into existing workflows. Providing opportunities for learning about PI prevention and program use may support the engagement of Chief CMs in the implementation process.

### Information-sharing tools

Considerations regarding information sharing tools were informed by challenges in multidisciplinary communication in the Outer Setting domain. In this domain, participants highlighted the need for approaches that facilitate accurate information sharing to reduce the associated burden. Specifically, CMs with social welfare backgrounds described difficulties in communicating with medical professionals, suggesting that gaps in information exchange may act as barriers to program implementation.

These findings suggest that it may be useful to consider developing tools that support efficient and accurate information sharing among professionals. Specifically, CMs with social welfare backgrounds experience challenges in communicating with medical professionals,^15^ and reducing this burden may contribute to a smoother exchange of information. Although the program includes guidance on collaboration with other professionals and family members, additional approaches to support information sharing may help address interprofessional communication gaps in the program.

With the increasing use of smartphones, digital tools, such as mobile applications, have been identified as practical approaches for PI follow-up. However, the availability and use of such tools in PI prevention remain limited.^37^ These findings suggest that a digital tool, such as a mobile application, which incorporates functions for recording PI risk assessment results (e.g., the PPRA-Home), sharing information with multidisciplinary teams, and accessing educational resources, may support interprofessional communication and information exchange in home care settings.

### Evidence generation

Considerations regarding evidence generation were informed by uncertainty regarding the program’s effectiveness in the Intervention Characteristics domain. In this domain, participants highlighted that the lack of established evidence regarding the clinical effectiveness and necessity of the PI prevention program incorporating the PPRA-Home may act as a barrier. Furthermore, the absence of immediate benefits or incentives for initiating the program were identified as additional obstacles to implementation.

Previous studies have demonstrated the reliability and validity of the PPRA-Home as a risk assessment scale.^25,26^ However, few studies have evaluated the effectiveness of the full PI prevention program that incorporates the PPRA-Home in actual clinical practice. Because none of the CMs in this study had prior experience using the program, they may not have been able to perceive or verify its benefits, which may have contributed to uncertainty regarding its effectiveness. Therefore, prospective studies may be needed to generate empirical evidence on the effectiveness of the intervention in real-world home care settings.

Extrinsic motivators, such as financial incentives, may contribute to initiating implementation because incentives can produce immediate effects. However, incentives alone may be insufficient to sustain implementation over time.^38^ These findings suggest that it may be useful to consider approaches that support a transition from extrinsic to intrinsic motivators to promote sustained implementation of the program in practice.

### Limitations

This pilot study has several limitations. First, the qualitative findings did not fully capture the process dimension of the CFIR framework because the participating CMs had no prior experience implementing the program in their actual practice. Furthermore, because the participants were recruited from a single facility, the findings may be influenced by the specific organizational culture and internal policies of that institution, which may not be representative of other home care settings. Second, the sample size was small, and the study was exploratory in nature; therefore, the generalizability of the results may be limited. Finally, strict COVID-19–related infection control measures in Japanese home care settings reduced face-to-face communication among CMs and between CMs and other professionals, which may have influenced participants’ perceptions and the data obtained. Further in-depth qualitative and mixed-methods studies may be needed to evaluate how best to implement the PI prevention program in routine home care practice.

## Conclusions

This exploratory qualitative study described CMs’ perceptions of potential facilitators and barriers to implementing a PI prevention program in home care settings. The findings should be interpreted cautiously because they were derived from a small sample recruited from a single facility. Future research may further examine the barriers and facilitators identified in this study and explore whether similar perceptions are observed in other settings and populations. Such research may contribute to a better understanding of the factors that influence the implementation of PI prevention programs in home care practice.

## Figures and Tables

**Table 1:** Characteristics of the study population

Characteristics	Overall (n=5)
Age in years, n (%)	
30–39	1 (20)
40–49	2 (40)
50–59	2 (40)
Sex, n (%)	
Male	1 (20)
Female	4 (80)
Level of education, n (%)	
Vocational college	1 (20)
University	4 (80)
Years since obtaining a care manager license, n (%)	
<3	1 (20)
5–9	1 (20)
≥10	3 (60)
Years of continuous employment in the current facility, n (%)	
<3	3 (60)
5–9	2 (40)
Professional background, n (%)^a^	Total responses=8
Social worker	4 (50)
Psychiatric social worker	1 (12.5)
Registered nurse	2 (25)
Certified public psychologist	1 (12.5)

Notes: ^a^ Some participants had more than one professional background.

**Table 2:** Facilitators and barriers of a pressure injury prevention program according to CFIR constructs

CFIR domains and constructs	Facilitators	Barriers
I. Intervention characteristics		
A. Intervention source		
B. Evidence strength and quality		• Unclear effect of intervention.
C. Relative advantage	• This is the only PI prevention program in home-based care settings in Japan.	• The program does not connect with the billing software for LTCI.
D. Adaptability	• Adaptability of the program reflects the existing risk factors for PI development. • Narrowing down cases. • Possibility of expanding the users to other professions.	• No specific methods for sharing the results with the multidisciplinary care team or the individual’s family.
E. Trainability	• Pilot study of the program to assess whether collaboration between care managers and other professionals is successful.	
F. Complexity	• The program is easy to use.	• How to collaborate with other professionals is unclear.
G. Design quality and packaging	• The structure of the program is simple and effective. • Structure to bring a change in the attitude of care managers.	• High number of target cases. The required time is extremely long. • Unclear when the program will be used. • Too frequent in practice. • Difficulty in information collection
H. Cost		
II. Outer setting		
A. Patient needs and resources	• Presence of a cooperative family. • Increased opportunity to think about PIs.	• Patients or their family refuse to support.
B. Cosmopolitanism	• Smooth communication with different professionals at different facilities.	• Difficulty in communication between care managers with medical backgrounds and those with social welfare backgrounds. • Fewer opportunities to communicate with care managers working at other facilities.
C. Peer pressure		
D. External policy and incentives	• Care benefit increments and financial support for PI prevention. • Policy improvements. • Expand the use of the program through education and increased awareness.	• Difficulty in collaborating with medical professionals. • Not fully educated on PIs at the time of receiving a care managers license. • Currently, there is limited awareness regarding PI prevention.
III. Inner setting		
A. Structural characteristics	• Improved contact with medical professionals. • Large number of cases transferred from hospitals to nursing homes.	• Lack of staff. • The perceived value of using the program is low in several facilities.
B. Networks and communications	• Easy access to consultation with other professionals	• Busy schedule hinders communication among care managers. • Lack of interest in cases other than their own.
C. Culture	• To schedule meetings to discuss PIs regularly. • Compliant and obedient to facilities. • Cooperation with each other in the care managers’ network.	• Excessive interference by a facility.
D. Implementation climate		
1. Tension for change		• Lack of perception of PI prevention among care managers. • Lack of tension for change regarding PI prevention.
2. Compatibility		• Lack of compatibility with the existing PI risk assessment tools.
3. Relative priority	• To have a common perception to use the program.	
4. Organizational incentives	• Expectations for increased remuneration for implementing the program.	
5. Goals and feedback		
6. Learning climate	• Opportunities for education and consultation based on good relationships between care managers.	
E. Readiness for implementation		
1. Leadership engagement	• Follow the instructions of their supervisor.	
2. Available resources		• Implementing the program increases daily workloads. • Lack of time because of overwork in daily practice.
3. Access to knowledge and information	• Regular workshops to acquire knowledge.	
IV. Characteristics of individuals		
A. Knowledge and beliefs about the intervention	• Successful experiences of PI preventive care.	• Limited perception of PI prevention. • Limited experience in consulting with other professionals about PI prevention. • Too many patients need the program.
B. Self-efficacy	• To have confidence in correctly implementing the program.	• Collaboration difficulties between care managers with medical backgrounds and those with social welfare backgrounds.
C. Individual stage of change		• Lack of interest in intervention and outcome.
D. Individual identification with organization	• To have a common perception of the program among facilities.	
E. Other personal attributes		• The excess work is incommensurate with the effectiveness of the intervention. • Lower perception of PI prevention among care managers with social welfare backgrounds. • Low mutual communication because of busy schedules. • Lack of experience in PI prevention.
V. Process		
A. Planning	• Planning and preparing for PI prevention. • Pilot study prior to the implementation of the program. • Incorporating the program into existing daily work schedules.	
B. Engaging		
1. Opinion leaders		
2. Formally appointed internal implementation leaders		
3. Champions		
4. External change agents		
C. Executing		
D. Reflecting and evaluating		

Abbreviations: CFIR, Consolidated Framework for Implementation Research; LTCI, Long-Term Care Insurance; PI, pressure injury.

## Adherence to reporting guidelines

As shown in Supplementary Material 1, we used the SRQR (Standards for reporting qualitative research) checklist found from the EQUATOR Network.

## Availability of data and materials

The datasets used and/or analyzed during the current study are available from the corresponding author upon reasonable request.
