# Supplementary material for: A preliminary qualitative exploration of perceived barriers and facilitators prior to implementation of a pressure injury prevention program in home care: perspectives of care managers in Japan
Source: Fujita Med J. 2026 Sep 25;12(4):304–11. doi: 10.20407/fmj.2026-001 (PMC13724190; doi:10.20407/fmj.2026-001)
Supplement: Supplementary file 1 [file fmj-12-304_s1.pdf]

Supplementary Material 1 SRQR Checklist

Item	Page
Item 1. Title: Concise description of the nature and topic of the study; identifying the study as qualitative or indicating the approach recommended	Page 1
Item 2. Abstract: Summary of key elements of the study	Page 1
Item 3. Problem formulation: Description and significance of the problem studied; review of relevant theory and empirical work	Pages 1-2
Item 4. Purpose or research question: Purpose and specific research objectives/questions	Page 2
Item 5. Qualitative approach and research paradigm: Approach (e.g., qualitative descriptive) and paradigm; rationale	Page 2
Item 6. Researcher characteristics and reflexivity	Page 2
Item 7. Context: Setting and contextual factors; rationale	Page 2
Item 8. Sampling strategy: How and why participants were selected; rationale	Page 2
Item 9. Ethical issues pertaining to human subjects	Page 2 and 6
Item 10. Data collection methods	Page 2
Item 11. Data collection instruments and technologies	Page 2
Item 12. Units of study	Page 2
Item 13. Data processing	Page 2
Item 14. Data analysis	Page 2
Item 15. Techniques to enhance trustworthiness	Page 2
Item 16. Synthesis and interpretation	Pages 2-6
Item 17. Links to empirical data	Pages 3-4
Item 18. Integration with prior work, implications, transferability	Pages 3-6
Item 19. Limitations	Page 6
Item 20. Conflicts of interest	Page 6
Item 21. Funding	Page 6

Supplementary Material 2

Interview guide for semistructured interviews for care managers

I. Intervention characteristics

Question 1. Do you think pressure injury prevention program (the program) interventions will help reduce the development of new pressure injuries in individuals receiving support/care under your charge?

Question 2. What are the likely facilitators and barriers to the adoption of the program? What are the most important things for care managers to adopt the program at in-home care support facilities in Japan?

Question 3. Are there any other interventions for preventing PI that are easier to implement than the program used in this study? What are the advantages and/or disadvantages of the program compared with any other intervention?

Question 4. What are your thoughts about the implementation of the program and the interventions in the future?

II. Outer setting

Question 5. To what extent do you think that the program meets the needs of the intended beneficiaries?

____

____

____

Question 6. How would the individuals under your care feel if the program was actually implemented in your practice?

____

____

____

Question 7. What are your requirements for successfully performing PPRA-Home assessment and implementing the program from the perspective of individuals receiving care?

____

____

____

Question 8. To what extent do you network with colleagues and people with similar titles, outside of the workplace? What type of things do you talk/discuss about with them?

____

____

____

Question 9. Please tell us which other facilities besides your own aim to prevent pressure injuries.

____

____

____

Question 10. Do you know the current policy and efforts of government and administrative agencies in preventing pressure injuries? What do you think they should do to promote pressure injury preventive measures in the in-home care support facilities?

____

____

____

III. Inner setting

Question 11. What is the impact of adopting the program on your facility? How do you think the program interventions can be facilitated or inhibited in your facility?

____

____

____

Question 12. What structural changes are required at your facility to conduct the program intervention? What are the most important things for the changes?

____

____

____

Question 13. Please tell us about your relationship with your colleagues and boss. Do you have daily communications with them within your facility? Do you discuss freely about business matters with them at your facility?

____

____

____

Question 14. Please tell us how you collect information on new topics or issues at your facility? How do you deal with such information?

____

____

____

Question 15. Is there anything you would like to comment on regarding your facility?

____

____

____

IV. Characteristics of individuals

Question 16. Please tell us what you know about pressure injury and its prevention.

____

____

____

____

Question 17. What do you think are the likely positive and negative effects of implementation of the program at your facilities?

____

____

____

Question 18. Do you feel confident in adopting the program into daily practice under your facility's current environment?

____

____

____

Question 19. How would your colleagues feel about adopting the intervention of the program?

____

____

____

V. Process

Question 20. What behavioral changes do you or your colleagues need to make to adopt the program at your facility?

____

____

____

Question 21. Are there any staff members who would be positive about adopting the program, other than yourself? If so, what type of professions would they belong to? How can these people facilitate the implementation of the program?

____

____

____

Thank you very much for your time and cooperation.
